# The Eye as a Transplantation Site to Monitor Pancreatic Islet Cell Plasticity

**DOI:** 10.3389/fendo.2021.652853

**Published:** 2021-04-23

**Authors:** Erwin Ilegems, Per-Olof Berggren

**Affiliations:** ^1^ The Rolf Luft Research Center for Diabetes and Endocrinology, Karolinska Institute, Stockholm, Sweden; ^2^ Diabetes Research Institute, Miller School of Medicine, University of Miami, Miami, FL, United States; ^3^ Lee Kong Chian School of Medicine, Nanyang Technological University, Singapore, Singapore; ^4^ Center for Diabetes and Metabolism Research, Department of Endocrinology and Metabolism, West China Hospital, Sichuan University, Chengdu, China; ^5^ School of Biomedical Sciences, Ulster University, Coleraine, United Kingdom

**Keywords:** pancreatic islet imaging, islet transplantation, anterior chamber of the eye, confocal microscopy, islet cell plasticity, beta cell, islet imaging, novel imaging methods

## Abstract

The endocrine cells confined in the islets of Langerhans are responsible for the maintenance of blood glucose homeostasis. In particular, beta cells produce and secrete insulin, an essential hormone regulating glucose uptake and metabolism. An insufficient amount of beta cells or defects in the molecular mechanisms leading to glucose-induced insulin secretion trigger the development of diabetes, a severe disease with epidemic spreading throughout the world. A comprehensive appreciation of the diverse adaptive procedures regulating beta cell mass and function is thus of paramount importance for the understanding of diabetes pathogenesis and for the development of effective therapeutic strategies. While significant findings were obtained by the use of islets isolated from the pancreas, *in vitro* studies are inherently limited since they lack the many factors influencing pancreatic islet cell function *in vivo* and do not allow for longitudinal monitoring of islet cell plasticity in the living organism. In this respect a number of imaging methodologies have been developed over the years for the study of islets *in situ* in the pancreas, a challenging task due to the relatively small size of the islets and their location, scattered throughout the organ. To increase imaging resolution and allow for longitudinal studies in individual islets, another strategy is based on the transplantation of islets into other sites that are more accessible for imaging. In this review we present the anterior chamber of the eye as a transplantation and imaging site for the study of pancreatic islet cell plasticity, and summarize the major research outcomes facilitated by this technological platform.

## Introduction

Diabetes mellitus is presently affecting large and growing segments of the population, especially the elderly, and represents a major socio-economic hurdle (1, 2). Constitutively high blood glucose is a symptom of this disease and causes a number of severe pathologies in multiple organs and cell types. Under normal conditions plasma insulin levels typically increase when blood glucose levels rise, and insulin serves as a signal for tissues throughout the body to take up glucose, thereby maintaining blood glucose levels within a narrow, physiologically optimal window. In the majority of cases, diabetes results from a progressive dysfunction in the supply of insulin secreted from the beta cells within the pancreatic islets, caused either by an insufficient number of these endocrine cells or by their failure to release adequate amounts of insulin in response to an increase in blood glucose concentration. In this respect it is necessary to further investigate the fine mechanisms linking glucose sensing to insulin release and how pancreatic islets can adapt to different circumstances to cope with varying insulin demands.

A major challenge in the longitudinal studies of islets is related to the fact that these small structures only represent about 1.5% of the total volume of the pancreas (3). Additionally, the pancreas itself is located deep in the abdomen between other organs and therefore not easily accessible for *in vivo* functional imaging. Although *in vitro* studies based on islets isolated from the pancreas brought significant advances to the understanding of islet biology, these are unfortunately inherently limited since they lack the many factors influencing pancreatic islet cell function *in vivo*, e.g. the effects of hormones secreted from a crosstalk with other organs such as liver, adipose tissue, brain, and gut (4, 5). Therefore, studies under the complex *in vivo* conditions present in the living organism are primordial for the longitudinal appreciation of islet function in health and disease.

A number of different advanced imaging techniques have been developed over the years for the study of pancreatic islets, with a particular focus on their ability to estimate beta cell mass, i.e. a volumetric measurement of insulin-positive cells (6). In particular, magnetic resonance imaging (MRI), positron emission tomography (PET), computer tomography (CT) and bioluminescence imaging (BLI) are the main imaging modalities used for *in vivo* noninvasive studies of islets in the pancreas. However, while these techniques offer appropriate imaging penetration for animal studies, they are still limited in terms of sensitivity and resolution and often require additional labeling for the detection of beta cells. Other techniques to image *in situ* pancreatic islets at higher resolution include optical projection tomography (OPT), light sheet fluorescence microscopy (LSFM), optical coherence tomography (OCT) and confocal microscopy, but require exteriorization or removal of the pancreas and therefore are not suitable for longitudinal imaging at single islet level. Alternatively, abdominal imaging windows have been installed with the aim of imaging islets *in situ* without delocalizing the pancreas (7, 8). While this solution allows for a repetitive optical assessment of individual islets, the number of imaging sessions is still limited and the procedure is technically very challenging.

Due to difficulties in imaging islets longitudinally in the intact pancreas, another strategy is based on transplantation of islets into other sites that are more accessible for imaging. One important aspect in this case, in addition to the possibility to image the islets at high resolution, is the proper engraftment and survival of transplanted islets. Revascularization and reinnervation are indeed primordial factors determining the outcome of islet transplantation due to their critical role in the maintenance of islet functionality and survival (9–11). In particular, intra-islet vessels require a fast formation for tissue oxygenation, and furthermore their endothelial cells have been shown to be involved in local interactions with beta cells that are of particular importance for islet function (12).

Multiple sites have been explored for islet transplantation (13). In particular the kidney subcapsular space, the spleen and the portal vein have been widely used for the *in vivo* evaluation of islet mass and function, albeit with varying levels of engraftment success (14, 15) and of accessibility for high resolution imaging. Another approach is based on the transplantation of pancreatic islets into the anterior chamber of the eye (ACE). Because of its optical and structural properties, the eye is optimally suited as a natural body-window for non-invasive and longitudinal imaging of single islet grafts and their vascularization. Islets transplanted into the ACE have been shown to be functional and various aspects of beta cell function and survival can be readily imaged in this environment. Furthermore, imaging islets at this site can be performed at relatively high speed and at resolutions allowing for single cell functional investigations, essential requirements for the assessment of beta cell heterogeneity and dynamics of intra-islet cellular communications (16). In the following we will review findings obtained by the use of the ACE as a transplantation/imaging site for longitudinal *in vivo* appreciation of pancreatic islet cell mass and function.

## The ACE as a Transplantation Site and the Cornea as a Natural Body Window for Imaging Pancreatic Islet Cells

The anterior chamber of the eye has been used as a transplantation site for about 150 years (17) and was shown in a number of studies to allow for the adequate engraftment of tissues from various origins, such as heart, brain, muscle, pituitary gland, liver, prostate, or tumors (18–23). The dense vascular network in the iris is contributing to rapid revascularization of the tissues (23, 24), providing essential nutrients and oxygenation for graft survival. In particular, pancreas tissue has been shown to benefit from proper survival after transplantation into the ACE (24–27). First seen as a convenient location for *in vivo* tissue culture and observation, this transplantation site has since been combined with high resolution microscopy for the assessment of islet morphology and function at individual islet and single cell level (28, 29). In addition to the benefits of this transplantation site for islet engraftment and imaging, the surgical procedure is particularly straightforward and is not causing pain nor affecting the vision of the recipient animal (30).

After their introduction into the ACE through a small perforation in the cornea, the islets attach to the iris, initiating their engraftment. A few days after transplantation, revascularization of the tissue starts by the appearance of large blood vessels, followed progressively by smaller capillaries (31). Approximately 4 weeks after transplantation islet grafts are completely vascularized and present a similar vascular density as compared to islets in the pancreas (28). Interestingly, it has been shown that the newly-formed vascular network originates from the combination of endothelial cells still residing within the transplanted islet and of endothelial cells emanating from the iris (32). It is important to note that, even when using islets or pseudoislets completely devoid of endothelial cells for transplantation, capillaries emanating from revascularization comprise fenestrations (32, 33), similar to those from islets in the pancreas and required for the optimal transit of compounds between endocrine cells and blood flow. The eye also benefits from a dense innervation, contributing to the supply of sympathetic and parasympathetic fibers to the transplanted tissue (18). Reinnervation of islets starts within a few days and reaches a plateau 3 months after transplantation, with a pattern dictated by the transplanted islet (34). Thus both the innervation, of importance for the modulation of insulin release (35), and the connection of the islet grafts to blood circulation are occurring in the ACE, a prerequisite for any comprehensive investigation of islet biology.

**Figure 1:**
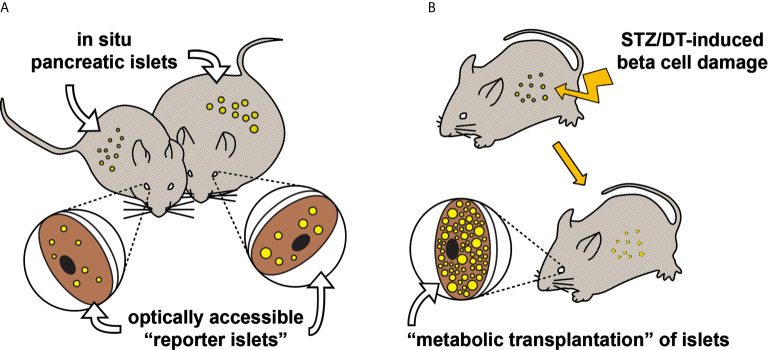
Two transplantation strategies for *in vivo* study of pancreatic islet function and plasticity. **(A)** Various aspects of pancreatic islet function and morphology have been shown to be mirrored in “reporter islets” in the eye (36). This illustrative example shows that changes occurring to the *in situ* pancreatic islets of an obese mouse (right), for instance beta cell hyperplasia, are similarly occurring to the islets transplanted into the ACE. **(B)** Mice rendered hyperglycemic, e.g. by streptozotocin (37) or diphteria toxin (38), can be recovered by “metabolic transplantation” of a large number of islets, taking over the function of damaged pancreatic islets.

There are basically two transplantation strategies, each giving different information on islet function (**Figure 1**). Firstly, the transplantation of a small number of islets, so-called “reporter islets”, will serve to indicate the function of islets in the pancreas without affecting overall blood glucose homeostasis. Secondly, the transplantation of a large number of islets (“metabolic transplantation”) into the ACE of mice rendered diabetic by the destruction of their pancreatic beta cells will regulate overall blood glucose homeostasis by secreting sufficient amounts of insulin in response to high glycemic levels, thereby taking over the role of islets in the pancreas. While the success of this latter procedure depends on the number of transplanted islets (37), it is interesting to note that only about 100 islets are required for complete recovery of blood glucose handling. Both transplantation strategies can be combined by the use of both eyes for transplantation, increasing the possibilities for scientific investigations.

A wide range of parameters related to islet function under normal and pathological conditions can be investigated by the use of the ACE as an *in vivo* transplantation/imaging platform (see **Table 1**). To start with, information on the morphology of the islet as well as its volume can be obtained by image acquisition of light scattering from islet cells. Indeed, secretory granules in beta cells are densely packed with crystallized insulin, forming microscopic spherical mirrors that are major contributors to the islet intrinsic scattering properties and permit to obtain volumetric information at cellular resolution without extrinsic labeling (39). Moreover, due to this biological origin, the intensity of light scattering is thereby directly indicative of insulin secretory capacity in islets. The recorded signal will thus be diminished both in cases of hypersecretion (36, 39) and of degranulation following autoimmune attack (53–55), thereby giving valuable insights into the functional status of transplanted islets.

**Table 1:** Morphological and functional parameters acquired in various studies from islets transplanted into the ACE.

Parameters indicative of islet function and morphology	References
*Beta cell mass*	(36, 39–41)
*Intra-islet vascular density and morphology*	(32, 42)
*Blood flow dynamics*	(43)
*Innervation*	(34)
*Biochemical composition*	(44)
*Beta cell-specific protein expression and localization*	(45)
*Beta cell [Ca^2+^]_i_ mobilization*	(33, 41, 46, 47)
*NAD(P)H response*	(40, 48)
*Insulin secretion status*	(39)
*Beta cell insulin resistance*	(49)
*Proteomic/metabolomic profile of islet microenvironment*	(50–52)
*Autoimmune-induced damage*	(53–57)
*Allorejection*	(51, 52, 58)
*Cell death*	(38, 55, 59)

Imaging strategies and probes for the investigation of cellular function are being continuously developed, many of which can be directly applied to the study of islet function *in vivo* after transplantation into the ACE. Many aspects, from glucose intake to insulin secretion, can now be readily imaged at various temporal resolutions (33, 39–41, 46–48). Probably the most studied signal in the beta cell signaling pathway is the change in cytoplasmic free Ca^2+^ concentrations ([Ca^2+^]_i_), for its central role in the secretion of insulin (60). This signal was first studied in islets engrafted into the ACE after intraocular incubation with a [Ca^2+^]_i_-sensitive fluorescent dye prior to *in vivo* activation with glibenclamide (28). Transgenic mice expressing genetically encoded fluorescent [Ca^2+^]_i_ indicators were later used to record beta cell responses to intravenous administration of glucose (41, 46, 47) and of vasopressin, an agonist for the beta cell expressed V1b receptor (a G protein-coupled receptor, GPCR) (33). Whereas the response to the GPCR agonist was immediate, glucose initiated a slightly slower increase in [Ca^2+^]_i_ that culminated about 50s after administration, followed by smaller oscillating signals that gradually disappeared in parallel to the subsequent decrease in blood glucose levels. These experiments showcase how *in vivo* experiments take into account the many factors (e.g. circulating glucose and hormone levels) whose complexity cannot be faithfully reproduced under *in vitro* conditions.

Although the ACE was previously thought to be an immune privileged site (61), as aforementioned it has been shown that islets could be subjected to autoimmune attack at this location (58). Moreover, the infiltration of T lymphocytes into islet grafts is supportive of the notion that the immune privilege is somehow lost during the transplantation/engraftment procedure, and that the ACE is well-suited for longitudinal *in vivo* studies of autoimmunity and allograft rejection. This also implies that rejection of islets originating from another genetic background/species will occur in unmatched recipient mice, but this can be circumvented by the use of immune-deficient mice. This is particularly attractive for the study of human islets and thereby for appreciating to which extent studies performed in rodents can be translated to humans. For instance human islets indeed differ from rodent islets in their endocrine cell content and architecture (62, 63) as well as in their innervation pattern and density (64), thereby displaying functional differences that can now be assessed *in vivo* using the ACE platform (65, 66). Furthermore, the possibility to study human islets longitudinally represents an important asset in that it allows to evaluate short- and long-term effects of pharmacological treatment strategies for diabetes. This has been done for evaluating long-term effects of the beta cell targeting antidiabetic drug liraglutide (65). In this study mice were rendered diabetic by administration of streptozotocin (STZ), followed by a “metabolic transplantation” of human islets. Mice treated systemically with liraglutide returned to normoglycemic levels in a shorter period of time than their controls, indicating a beneficial short-term effect. However, upon prolonged treatment the transplanted islets became dysfunctional, unable to maintain sufficient insulin release to sustain normal glucose levels. These findings indicate that an excessive stimulation of beta cells with liraglutide leads to beta cell exhaustion and failure, which may be of immediate relevance for the outcome of long-term treatment of type 2 diabetes patients with this kind of drugs.

It is therefore possible to study in detail the effect of a pharmacological treatment on beta cell function and survival by systemic administration using this methodological platform. Interestingly, due to the specific location of the transplanted islets it is also possible to proceed with topical drug delivery, targeting more directly the engrafted tissue. In particular, compounds can be administrated locally by the application of eye drops (66) or by the slow release of compounds from co-transplanted micro-containers (67), thereby circumventing potential systemic adverse effects and reducing the overall treatment dosage. Finally, in addition to the advantages of using the ACE for islet transplantation and assessment, this accessible site can be used for analyzing compounds accumulating in the islet micro-environment (50–52). Microliter-size aqueous samples can be obtained in the immediate vicinity of the engrafted tissue, allowing for the analysis of islet-related metabolites and proteins. In particular, this strategy has been used with the aim to define early predictive markers of type 1 diabetes by detecting changes in the metabolic profile (50), and to predict the risk of allograft rejection to allow for a timely therapeutic intervention (51, 52). As a whole, these methodologies using the ACE platform are perfectly suited for the study of islet plasticity in health and disease under *in vivo* conditions.

## Longitudinal Imaging of Pancreatic Islet Cell Plasticity: Validation Studies and Scientific Advances

Under various physiological circumstances islets can display a certain degree of adaptation, for instance an increased demand for insulin can lead to an increase in beta cell mass (68). The modulation of beta cell function, either in some individual beta cells within islets or in the entire population of beta cells, can also serve as an adaptive biological mechanism of importance for the maintenance of normoglycemia. Both physiological and pathological states may cause changes in these adaptive mechanisms, which can lead to the incapacity in maintaining normoglycemia and to the development of diabetes. One of the major assets of the ACE as a transplantation/imaging platform is to allow for the investigation of islet plasticity over a period of several months, and thereby for the study of the diverse adaptive procedures and circulating factors involved in the regulation of beta cell mass and function.

### Increase and Decrease in Beta Cell Mass

Longitudinal changes in beta cell mass have first been reported for islets transplanted into the ACE of the ob/ob mouse model (36). This mouse model lacks functional leptin and therefore its uncontrolled appetite quickly leads to obesity. The study showed that islets transplanted into the eye rapidly grow as a consequence of beta cell hyperplasia, similarly to what has been previously been reported for islets in their pancreas (69). Islet volume doubled in a period of one month as a consequence of beta cell proliferation, a result corroborating with the impressive beta cell mass expansion seen in the pancreas by OPT (70). Although the islets transplanted into the ACE cannot fully represent the entire range of islets that are existing in the pancreas, due to the different size distribution among isolated islets as compared to *in situ* pancreatic islets (33, 59), we could show that reporter islets in the ACE served as a representative sample that displayed identical beta cell replication as compared to *in situ* pancreatic islets. Also, the dilation of intra-islet blood capillaries, suggested to be due to increased parasympathetic innervation and endothelial nitric oxide production in ob/ob mouse islets (71), was observed in the reporter islets. Interestingly, both islet growth and enlarged capillaries were shown to be dependent on the recipient mouse and not on the origin of the transplanted islets, illustrating the importance of circulating factors for these morphological phenotypes. In addition, reporter islets in the ACE could be used to visualize the efficiency of a treatment. For example, by daily intraperitoneal injections of leptin, the appetite of ob/ob mice was reduced and the growth of their islets was halted, both in the ACE and *in situ* in the pancreas (36). Reporter islets were similarly used in other studies for investigating changes in beta cell mass when mice were fed a high fat diet (HFD) (40, 41). Interestingly, islet growth was relatively modest under HFD-induced prediabetes conditions (islet volumes were doubled after a period of 4 month on HFD), and it was shown that the major compensatory mechanism to cope with insulin resistance was a change in islet function to increase insulin secretion from individual beta cells (41).

A decrease in beta cell mass has also been documented with islets transplanted into the ACE using various mouse models for the study of diabetes. For instance, autoimmune destruction of beta cells was assessed in the NOD mouse model, showing both the infiltration of fluorescently-labelled immune cells and the progressive and rapid destruction of islet cells following diabetes onset (55). Interestingly, this study showed that during the short pre-diabetes period, islet volumes were temporarily increased and their scattering properties were five-fold reduced, indicative of insulin hyper-secretion (39). These findings show that reporter islets in the ACE allow for the early detection of the pathogenesis of type 1 diabetes, with the potential to provide a therapeutic intervention on a timely manner, before the full development of diabetes. Beta cell ablation was also monitored in other widely used mouse models for the study of diabetes and hyperglycemia. The kinetics and extent of toxin-mediated beta cell destruction were monitored both in the RIP-DTR (38) and in the STZ-diabetic models (59), and validated by OPT image analysis of islets in the pancreas. The destruction of islets in the RIP-DTR mouse model was almost total after only a few days, due to an immediate effect of the toxin fully occurring during the day of administration and leading to the inhibition of beta cell protein synthesis (72), and subsequently to beta cell death. Interestingly, glucose handling started to be affected only 2 days after toxin administration despite a discontinuation in the expression of all proteins required for glucose sensing/metabolism and insulin granule secretion. This fact highlights the over-capacity in terms of pancreatic islet function, displaying a surprising functional reserve for the maintenance of blood glucose levels. In mice rendered hyperglycemic by STZ, beta cell mass was much less affected as compared to the RIP-DTR mouse model. Beta cell ablation occurred mainly during the first week after STZ administration, and mice became hyperglycemic with more than half of their beta cell mass remaining (59). This study demonstrated that STZ was mainly affecting the function of pancreatic beta cells rather than beta cell mass. Interestingly, when proceeding with a metabolic transplantation of islets into the ACE following STZ-induced hyperglycemia, supporting the remaining beta cells in the islets within the pancreas in their efforts to regulate blood glucose homeostasis and thereby reducing their metabolic stress, they partially recovered in terms of function and maturity. Jointly, the results from this study indicate that hyperglycemia in itself sustains a negative feedback loop restraining the recovery of islet function, and highlight the impressive plasticity of the endocrine pancreas, even after STZ-induced damage (59).

### Functional Plasticity of Pancreatic Islets

Although plasticity in beta cell function can be indirectly inferred by the acquisition of physiological parameters, different imageable indicators can be used to more specifically report on beta cell function using islets engrafted into the ACE. For instance in mice fed a HFD, beta cell [Ca^2+^]_i_ dynamics were monitored during the development of prediabetes by the use of the GCaMP3 fluorescent indicator (41). In this study it was shown that glucose-induced increase in beta cell [Ca^2+^]_i_ was already reduced after one week of HFD, and basal non-stimulated levels of [Ca^2+^]_i_ were increased progressively to a significant level two months after introduction of the diet. Combined with longitudinal imaging of beta cell mass, these results demonstrate that alterations in beta cell function and efficacy in terms of glucose-induced insulin release prevail over the increase in beta cell mass to compensate for insulin resistance in HFD-induced prediabetes (41). This study further showed that, after 4 months of HFD, beta cell [Ca^2+^]_i_ responses to glucose could be reverted by refeeding mice a normal diet for 2 weeks only, in parallel with a reversal of the prediabetes status. Altogether these findings support the notion that beta cell function should be the primary target for the treatment of diet-induced diabetes rather than beta cell mass.

Glucose-induced [Ca^2+^]_i_ responses were later investigated at the single beta cell level in islets transplanted into the ACE of ob/ob mice (46). While intravenously injected glucose was shown to reach all beta cells simultaneously, both in control and ob/ob mice, this resulted in the activation of only about 20% beta cells in the hyperglycemic ob/ob mouse as compared to about 80% in control mice, at 2 months of age. In another study using islets transplanted into the ACE, it was shown that the number of responding beta cells depends on their connectivity within the islet, which under normal conditions increases when glucose levels are elevated (47). This implies that beta cells are less coordinated in ob/ob as compared to control mice. Interestingly, the percentage of responding beta cells increased over time and became identical to control mice when they reached 10 months of age (46). These findings illustrate the functional plasticity occurring in islets of the ob/ob mouse over time, and how reporter islets in the ACE allow for the investigation of beta cell functional heterogeneity at single islet level.

Although beta cell connectivity was increased in the 10-month-old ob/ob mice, not all aspects of their islet function were improved. Indeed, it was shown using vibrational microscopy and multivariate analysis, that reporter islets transplanted into the ACE of ob/ob mice had a higher content of collagen (44). This technique allows to register changes in the chemical composition of tissues and in this case reported on an increase in blood vessel fibrosis in ob/ob mouse islets. Electron microscopy studies confirmed the strong deposit of collagen fibers surrounding intra-islet endothelial cells, both in islets in the pancreas and in islets engrafted into the ACE. Fibrosis of islet blood vessels has been similarly detected in old mice by *ex vivo* analysis of islets transplanted into the ACE (31), altogether pointing to a natural, progressive and irreversible increase of fibrosis over time that is exacerbated by diabetic conditions.

## Discussion

Over the years, *in vivo* imaging of islets transplanted into the ACE has proven to be a remarkably valuable tool for the study of pancreatic islet biology and plasticity. Despite the numerous scientific findings obtained from its use, a few limitations have however to be acknowledged. First and foremost, islets engrafted into the ACE might not reflect in all respects the function and plasticity of islets *in situ* in the pancreas, simply due to their different location. For instance, even though engrafted islets are properly innervated both by sympathetic and parasympathetic neurons, they likely are not connected by circuits emanating from the hypothalamic regions as is the case for *in situ* pancreatic islets (73). The potentiating effect of light on insulin secretion from islets engrafted into the ACE (34) is indicative of connections from the grafts to the visual cortex instead, as demonstrated for other tissues engrafted into the ACE (18). Also, the relative location of the engrafted islets with regard to other organs contributing to blood glucose homeostasis is different from that of islets in the pancreas, which may have functional implications. For example, while the liver is the first organ exposed to insulin released from islets in the pancreas and is the main contributor to insulin clearance (74–77), insulin released from engrafted islets will first encounter other organs before reaching the liver at a progressively diminished concentration. Although it would imply that islets transplanted into the ACE might not utterly mirror *in situ* pancreatic islets in their functionality, this very fact can potentially shed light on the importance of a stepwise presentation of insulin at different concentrations to different organs for the normal function of the endocrine pancreas. In this respect, it is interesting to note that only about 100 islets are required to be transplanted into the ACE of a mouse devoid of functional *in situ* pancreatic islets (37, 59, 66), which suggests a seemingly more efficient insulin signaling between engrafted islets and peripheral organs. Finally, potential endocrine-exocrine interactions in the pancreas, of beneficial or detrimental nature for islet function, will not apply to islets transplanted into the ACE. It is therefore of importance to keep these differences in mind when studying islets engrafted into the ACE, and possibly even make use of these to reveal specific aspects related to the function of *in situ* pancreatic islets and to inter-organ communication in health and disease.

Contrasting to the use of the ACE to study transplanted islets as “reporters”, mirroring as accurately as possible pancreatic islet function and plasticity, the ACE can also be a valuable transplantation site to investigate in detail the role of specific genes and signaling pathways on islet function. For instance, islet-expressed ApoCIII was shown by longitudinal *in vivo* imaging and *ex vivo* functional assessment to have a major role in islet growth and signaling (48). Also, transplantation of islets from different species (mouse, human, monkey) into the ACE of mice revealed that intra-islet paracrine interactions are major determinants of the resting glycemic set point *in vivo* (66). The accumulation of such discoveries can serve as the basis for the establishment of all fundamental parameters required for proper islet function. Moreover, and in addition to establishing this optimal repertoire, a further possible “enhancement” of islet function could prove to be beneficial for future clinical transplantation, which suffer to this day from poor islet quality and survival (78). This transplantation and imaging platform has indeed already been successfully used to assess functional enhancement of synthetically engineered islet-derived pseudoislets (33). It could similarly be used in the relatively near future to study potentially beneficial effects of modifying GPCR signaling pathways and/or intra-islet paracrine interactions (79), incorporating supporting structures or accessory cells in islets (80), or using stem cells as a new source of mature islet cells (81–83), altogether supporting the development of promising strategies to improve the outcome of clinical transplantations. In conclusion, the ACE has been a remarkable research platform that is being adopted by an increasing number of scientists for the study of islet function and survival, and without doubt will continue to support novel findings in the field of islet biology and innovative therapeutic approaches for diabetes treatment.

## Author Contributions

EI wrote the review article. P-OB revised and edited the review article. All authors contributed to the article and approved the submitted version.

## Funding

Own work discussed in this review was supported by funding from Karolinska Institutet, the Strategic Research Program in Diabetes at Karolinska Institutet, the Swedish Research Council, the Novo Nordisk Foundation, the Swedish Diabetes Association, the Family Knut and Alice Wallenberg Foundation, Diabetes Research and Wellness Foundation, the Stichting af Jochnick Foundation, the Family Erling-Persson Foundation, Berth von Kantzow’s Foundation, ERC-2018-AdG 834860 EYELETS, the European Union’s Seventh Framework Programme under grant agreements No 289932 and 613879, and the European Diabetes Research Programme in Cellular Plasticity Underlying the Pathophysiology of Type 2 Diabetes.

## Conflict of Interest

P-OB is founder and CEO of Biocrine AB. EI is consultant for Biocrine AB.
